# Hyaluronic Acid Hydrogel Inhibits Autophagy Through the miR‐181a‐5p/ATG5 Molecular Axis to Promote the Adipogenic Differentiation of Adipose‐Derived Stem Cells

**DOI:** 10.1155/sci/6997518

**Published:** 2026-06-30

**Authors:** Fengshan Gan, Fan Zheng, Qingzhu Zhou, Boyan Liu, Bin Yang, Lianzhu Ou, Wenli Huang, Xinxin Yang, Zhuo Gong, Yunyu Xiong, Xian Zhao, Jia He

**Affiliations:** ^1^ Plastic and Cosmetic Surgery Department, The First People’s Hospital of Kunming, Kunming, 650200, Yunnan, China

**Keywords:** adipogenic differentiation, adipose-derived stem cells, ATG5, autophagy, hyaluronic acid hydrogel, miR-181a-5p

## Abstract

**Background:**

Soft tissue defects refer to defects in or damage to human soft tissue, which may affect the appearance, function, and health of the human body and cause physical and psychological distress to the patient. Hyaluronic acid (HA), a fillable material in the field of plastic medicine, could play a crucial role in reconstructing soft tissues and enhancing the adipogenic differentiation of adipose‐derived stem cells (ADSCs).

**Methods:**

We isolated ADSCs from human subcutaneous adipose tissue and cultured them in HA hydrogel at a density of 2.1 × 10^6^/mL, followed by subcutaneous injections of ADSCs (0.2 mL, 1 × 10^7^ cells/mL with 0.2 mL of DMEM) and HA hydrogel (0.2 mL) into the dorsal side of nude mice. The expression of related genes and proteins was detected using RT‒qPCR and western blotting. The adipogenic differentiation of ADSCs and adipogenesis in nude mice were assessed using CCK‐8, Oil Red O, and HE staining.

**Results:**

The results revealed an increase in the levels of miR‐181a‐5p and adipocyte differentiation‐related proteins (PPARγ, C/EBPα, FABP4, and adiponectin) and a decrease in the levels of autophagy‐related proteins (LC3II/I, Beclin‐1, and ATG5) in ADSCs cocultured with HA hydrogel. The adipogenic differentiation ability of ADSCs was enhanced. From a mechanistic standpoint, HA hydrogel inhibited the expression of ATG5 by promoting the expression of miR‐181a‐5p, thus inhibiting autophagy, promoting the adipogenic differentiation of ADSCs, and promoting the formation of adipose tissue in vivo.

**Conclusions:**

Our findings suggest that a combined culture of ADSCs and HA hydrogel may be a new method to enhance the adipogenic differentiation of ADSCs.

## 1. Introduction

Plastic surgery has been dedicated to addressing health and esthetic concerns caused by soft‐tissue defects through various transplants and fillings. Autologous fat grafts (AFTs) are one of the materials used in soft tissue repair and reconstruction, fat grafts are considered ideal soft tissue fillers because of their biocompatibility, lack of immunogenicity, and availability and are widely used in plastic surgery [1]. However, after AFT transplantation, the tissue is prone to insufficient blood supply, which leads to the absorption of the fat‐transplanted tissue and the apoptosis of adipocytes [2]. Moreover, the processes of breast augmentation were limited by the reabsorption of fat, necessitating numerous filler injections for optimal outcomes. Therefore, finding new fat‐filling methods and improving the survival rate of transplanted cells are ways in which the filling effect can be controlled in plastic surgery.

The emergence of tissue engineering and regenerative medicine offers new promise in soft tissue repair, and the application of stem cells is a new therapy in plastic surgery [3]. Adipose‐derived stem cells (ADSCs) are a group of cells derived from adipose tissue with potential for differentiation and can be used in tissue engineering regenerative medicine [4]. ADSCs have obvious advantages in promoting the early neovascularization, the proliferation and differentiation of fat cells, and regeneration and renewal of transplanted adipose tissues [5]. For example, ADSCs, as autologous stem cells, can alleviate immune rejection, promote damage repair, restore the skin barrier, and improve photoaging symptoms [6]. However, the transplantation of ADSCs is affected by the in vivo microenvironment, making it difficult for ADSCs to perform their multiple functions [7]. For ADSCs to function optimally, a suitable environmental niche needs to be established. Therefore, it is crucial to search for protective carriers for ADSCs. Hyaluronic acid (HA) has excellent biocompatibility, degradability, and non‐immunogenicity and plays an important role in cell migration, adhesion, proliferation, and differentiation [8]. Currently, HA is widely used as a filler material in plastic surgery. Research has indicated that BMP‐14‐modified ADSCs encapsulated in HA hydrogels can accelerate the repair of cartilage defects in rabbits [9]. However, the role of HA hydrogels in the adipogenic differentiation of ADSCs remains largely unclear. Therefore, in this study, ADSCs were cultured in HA hydrogels, and the potential molecular mechanisms by which HA hydrogels affect the adipogenic differentiation of ADSCs were explored.

Autophagy plays a key role in regulating the differentiation of pre‐fat tissue into mature tissue [10, 11], indicating that autophagy plays a potential role in promoting the recovery of soft tissues by ADSCs. As one of the key regulators of autophagy, ATG5 is involved in the formation of autophagosomes and enhances autophagy [12]. Crucially, ADSCs secretion can alleviate the autophagic injury of hepatocytes by regulating the expression of ATG5 [13]. Therefore, we speculate that ATG5‐mediated autophagy may play an important role in the adipogenic differentiation of ADSCs.

Furthermore, numerous studies have demonstrated that microRNAs (miRNAs) can regulate the adipogenic differentiation of ADSCs. miRNAs are small noncoding RNAs that are widely present in eukaryotes. By pairing with the complementary sequence of the 3^′^UTR on the target mRNA transcript, miRNAs participate in the regulation of posttranscriptional gene expression [14]. A previous study revealed that miR‐145‐5p and miR‐148a‐3p are involved in the adipogenic differentiation process of ADSCs [15]. Furthermore, studies have confirmed that miR‐181a‐5p is highly expressed in ADSCs and their exosomes [16, 17]. A recent study revealed that HA equipped with exosomes rich in miR‐181a‐5p can improve hepatic stellate cell (HSC) fibrosis [18]. Ouyang et al. [19] reported that miR‐181a‐5p can enhance differentiation and fat production of 3T3‐L1 preadipocytes. These findings reveal that miR‐181a‐5p may play a key role in the biological functions of ADSCs. However, whether miR‐181a‐5p affects the role of HA hydrogels in the adipogenic differentiation of ADSCs is currently unclear. In addition, studies have shown that ATG5 is a direct target of miR‐181a‐5p and that miR‐181a‐5p can promote autophagy in endothelial cells [20]. However, the role of the miR‐181a‐5p/ATG5 regulatory axis in ADSCs cultured with HA hydrogels has not yet been reported.

On the basis of the above analysis, this study aimed to explore whether HA hydrogels affect the autophagy and adipogenic differentiation of ADSCs by regulating the miR‐181a‐5p/ATG5 molecular axis. The results of this study are expected to provide new strategies and intervention targets for clinical fat‐filling regimens.

## 2. Materials and Methods

### 2.1. Isolation of Primary Human ADSCs

Human primary ADSCs were isolated from the subcutaneous adipose tissue of the abdomen of a volunteer (aged 25 years) in the Department of Plastic and Cosmetic Surgery at our hospital. Informed consent was obtained from all individual participants prior to any procedures related to the research. Approximately 20 mL of the liposuction‐derived lipid fraction was subjected to multiple PBS washes to eliminate discernible fibers and blood vessels in the fat tissue. These samples were then sliced into delicate tissue segments, spun at 1000 rpm for 5 min to separate the middle layer of fat particles, and then moved to a 50 mL tube for centrifugation. Type I collagenase (Sigma‒Aldrich, USA) was added in equal parts, and the mixture was digested in a steady‐temperature water bath at 37°C for ~2 h. Throughout this time, the centrifuge tubes were shaken by hand. Digestion was stopped when the adipose tissue became fine and sandy. The mixture was subsequently centrifuged at 1500 rpm for 5 min, after which the supernatant was removed. An equal amount of DMEM (Gibco, USA) was added for neutralization, the mixture was centrifuged at 1500 rpm for 5 min, and the supernatant was then discarded. An appropriate amount of DMEM was used to suspend the cells. The cell density was adjusted to 1–2 × 10^4^ cells/mL, and the cells were aliquoted into T25 culture flasks and then incubated in a 5% CO_2_ incubator at 37°C. Observations of the cells were conducted daily, and the medium was changed for the first time at 24 h to remove nonadherent cells, followed by a change every 2–3 days.

### 2.2. Culture and Transfection of ADSCs

Preparation of the HA hydrogel: The HA hydrogel was prepared according to previous methods [21]. In brief, the HA hydrogel was prepared by mixing CMHA‐S and HP‐DTPH solutions with Gtn‐DTPH solution at a volume ratio of 1:1 and 0.056% (w/v) polyethylene glycol diacrylate (PEGDA) crosslinking agent at a volume ratio of 4:1. The hydrogel was transferred into 96‐well plates with glass bottoms and allowed to gel at 37°C for an hour.

Cell culture: The separated human ADSCs were grown in HA hydrogels at a density of 2.1 × 10^6^ cells/mL. Then, an adipogenic differentiation medium was added, and the cells were incubated in a 5% CO_2_ incubator at 37°C. The culture medium was changed every 2 days. After 21 days of induction, adipogenesis and adipogenic markers were detected. To explore the effect of autophagy on the adipogenic differentiation of ADSCs, we added 100 nM of the autophagy activator rapamycin (Aladdin, Shanghai, China) to the ADSCs culture medium and incubated it for 24 h.

For cell transfection, human ADSCs were cultured overnight in 24‐well plates. Upon reaching a cell density of 60%–70%, NC inhibitor, miR‐181a‐5p inhibitor, NC mimic, miR‐181a‐5p mimic, OE‐NC, and OE‐ATG5 were added to ADSCs in groups following the Lipofectamine 3000 reagent guidelines (Invitrogen, Grand Island, NY, USA). The cells were cultured in an incubator at 37°C and 5% CO_2_ for 48 h, after which the transfection efficiency was measured.

### 2.3. Fat Transplantation Model in Nude Mice

Thirty female BALB/c nude mice (weighing 16–18 g) aged 6–8 weeks were obtained from the Animal Experiment Center of Kunming Medical University for this study. Following 1 week of adaptive feeding, the test animals were randomly divided into three groups: the normal control group, the ADSCs group, and the HA hydrogel + ADSCs group. Nude mice received subcutaneous injections of ADSCs (0.2 mL, 1 × 10^7^ cells/mL, and 0.2 mL of DMEM) or a mixture of ADSCs and HA hydrogel (0.2 mL) using a 1 mL syringe and a 19G needle under analgesia. Eight weeks after subcutaneous injection, the nude mice were euthanized, and the newly formed adipose tissues were collected to determine the relevant indicators.

### 2.4. RT‒qPCR

Total RNA was extracted from human ADSCs via the TRIzol reagent (Invitrogen, 15596026). cDNA synthesis from RNA was achieved through reverse transcription using a first‐strand cDNA synthesis kit (GenNode, China), and RT‒qPCR was performed using a SYBR Green real‐time PCR kit (Solarbio, China). Using U6 as a reference gene, the relative gene expression was calculated via the 2^−ΔΔCt^ method. The primer sequences can be found in Table 1.

**Table 1:** Primer sequences.

Target	Sequence (F: forward primer; R: reverse primer) (5′‐3′)
miR‐181a‐5p	F: CGAACATTCAACGCTGTCG R: AGTGCAGGGTCCGAGGTATT
U6	F: CTCGCTTCGGCAGCACA
	R: AACGCTTCACGAATTTGCGT

### 2.5. Western Blot Analysis

Total proteins were extracted from treated human ADSCs using a cell lysis solution (Beyotime, China) supplemented with 1 mM phenylmethylsulfonyl fluoride and a protein phosphatase inhibitor (1x; Solarbio, China). The protein concentration was measured using a NanoDropTM 2000/2000c spectrometer (Thermo Fisher Scientific; Wilmington, DE). After mixing with 5x concentration buffer (Beyotime, China), the samples were boiled at 100°C for 10 min, followed by storage at −80°C. The protein sample (100 μg) was separated via SDS‒PAGE and then transferred onto PVDF membranes (Millipore, Billerica, MA). Following a 1‐h blockage with 5% milk, the samples were incubated overnight at 4°C with the primary antibody. The membrane was subsequently incubated with a secondary antibody (1:4000, ab97051, Abcam, UK) at room temperature for 1 h. The images were captured using a FluorChem E system (ProteinSimple, San Jose, CA) and analyzed by ImageJ software.

The primary antibodies used included the following: PPARγ (1:1000, ab316982, Abcam, UK), C/EBPα (1:1000, ab140479, Abcam, UK), FABP4 (1:1000, ab92501, Abcam, UK), adiponectin (1:1000, ab22554, Abcam, UK), LC3B (1:2000, ab192890, Abcam, UK), ATG5 (1:1000, ab108327, Abcam, UK), Beclin‐1 (1:1000, ab302669, Abcam, UK), and GAPDH (1:1000, ab181602, Abcam).

### 2.6. Flow Cytometry

Analysis of the human ADSC phenotype was conducted using flow cytometry with a Partec flow cytometer. The following monoclonal antibodies were used: CD29 (1:200, BioLegend, USA), CD34 (1:200, BioLegend, USA), CD44 (1:200, BioLegend, USA), CD45 (1:200, BioLegend, USA), CD90 (1:200, BioLegend, USA), and CD105 (1:200, BioLegend, USA). Newly separated human ADSCs were incubated for 20 min with 200 μL of the primary antibody in PBS, followed by a 2‐min dark incubation with 200 μL of the FITC‐linked secondary antibody. After two washes, the cells were reconstituted in 500 μL of PBS for examination.

### 2.7. CCK‐8

Human ADSCs (5 × 10^3^ cells/well) were inoculated in 96‐well plates and cultured in an incubator at 37°C and 5% CO_2_ for 24 h. Then, 10 μL of CCK‐8 reagent (C0037, Beyotime, China) was added to each well. After incubation for 2 h, the absorbance value of each well was measured at 450 nm using a microplate reader (Thermo Fisher Scientific, MA, USA).

### 2.8. Oil Red O Staining

ADSCs were fixed with 4% paraformaldehyde for 30 min and then washed twice with PBS. Fixed adipose tissue was encapsulated by O.C.T. and then cut into 5 μm sections. Then, isopropanol was added to the cell and tissue samples. Oil Red O staining solution was added, and the samples were incubated in the dark for 15 min, washed with double‐distilled water, stained with hematoxylin for 1 min, and observed and photographed under a microscope (Nikon, Japan).

### 2.9. Immunofluorescence Staining

The cells were washed twice with PBS, fixed with 4% paraformaldehyde for 30 min, permeated with 0.5% Triton X‐100 for 10 min, and blocked with bovine serum albumin for 1 h. Then, the cells were incubated overnight at 4°C with a primary antibody against LC3B (1:200, ab192890, Abcam, UK). The cells were subsequently incubated with the corresponding secondary antibody for 1 h and stained with DAPI. Ultimately, the dyed cells were examined, and photographs were taken using a fluorescence microscope (400857, Nikon, Japan).

### 2.10. Dual‐Luciferase Experiment

The specific binding sites of miR‐181a‐5p and ATG5 were predicted via a bioinformatics website (http://starbase.sysu.edu.cn/). The ATG5 3^′^‐UTR containing the miR‐181a‐5p binding site was cloned and inserted into the pGL3 vector (Promega) to construct the wild‐type ATG5 vector (ATG5 WT). The generation of mutant ATG5 vectors (ATG5 MUT) was accomplished using a site‐directed mutagenesis kit (Stratagene, USA). The WT or MUT vectors were cotransfected with the miR‐181a‐5p mimic or negative control into 293T cells using Lipofectamine 3000. Following a 48‐h period, the activity of luciferase was identified using a dual‐luciferase reporter assay system (Promega).

### 2.11. HE Staining

The adipose tissue sections were dewaxed in xylene for 5 min, rehydrated in gradient alcohol, and soaked in ddH_2_O for 2 min. The samples were subsequently stained with hematoxylin (Solarbio, China) for 15 min, followed by two washes with tap water, and then stained with eosin for 2 min. After the sections were dehydrated, made transparent, and sealed with neutral resin (Sigma‒Aldrich, USA), the stained adipose tissue was observed under a microscope (Nikon, Tokyo, Japan).

### 2.12. Immunohistochemical Staining

The adipose tissue sections of the nude mice were dewaxed and rehydrated, after which antigen retrieval was performed in a 0.01 M citrate buffer (pH 6.0). The sections were incubated overnight at 4°C with antibodies against FABP4 (1:200), PPARγ (1:300), ATG5 (1:200), and CD31 (1:200), and an immunoassay was performed with an HRP‐conjugated secondary antibody and DAB agent used in immunodetection. The stained sections were observed under a microscope (400857, Nikon, Japan).

### 2.13. Statistical Analysis

The experimental data are presented as the means ± standard deviations (means ± SDs). Analysis and visualization of the data were conducted using GraphPad Prism 7. The Shapiro–Wilk test was used to evaluate the normality of the experimental data. A *t* test was used to compare two groups, whereas one‐way ANOVA or two‐way ANOVA with Fisher’s LSD post‐hoc test was used to compare multiple groups. *p* < 0.05 indicated that the difference was statistically significant.

## 3. Results

### 3.1. HA Hydrogels Promoted the Survival and Adipogenic Differentiation of ADSCs and Inhibited Autophagy

First, we detected the effect of the HA hydrogel on ADSCs. Flow cytometry revealed that in isolated ADSCs, the levels of the surface markers CD29, CD105, CD44, and CD90 were 99.5%, 99.9%, 99.8%, and 99.3%, respectively, while the percentages of CD34‐ and CD45‐positive ADSCs were 0.01% and 0.11%, respectively (Figure 1A). Compared with that in the ADSCs group, the survival rate of ADSCs in the HA hydrogel and ADSCs group was markedly greater (Figure 1B), and the adipogenic ability was also markedly increased (Figure 1C). The expression of proteins related to adipocyte differentiation was subsequently detected. The findings revealed a notable increase in the expression of PPARγ, C/EBPα, FABP4, and adiponectin compared with that in the ADSCs group following HA hydrogel and ADSCs coculture (Figure 1D). These findings revealed that ADSCs successfully differentiated into adipocytes. Earlier research has indicated that autophagy may hinder the adipogenic differentiation of ADSCs [22]. Consequently, we detected the expression of proteins linked to autophagy and demonstrated that the HA hydrogel and ADSCs coculture groups had notably reduced levels of LC3II/I, Beclin‐1, and ATG5 (Figure 1E). Immunofluorescence analysis also revealed that the HA hydrogels markedly reduced the LC3B expression in ADSCs (Figure 1F). These findings revealed that the HA hydrogel promoted the survival and adipogenic differentiation of ADSCs and inhibited autophagy.

Figure 1HA hydrogels promoted the survival and adipogenic differentiation of ADSCs and inhibited autophagy. (A) Identification of ADSCs surface markers through flow cytometry. (B) ADSCs viability was detected by the CCK‐8 assay. (C) ADSCs adipogenic differentiation ability was detected via Oil Red O staining; scale bar: 50 μm. (D) Proteins linked to adipocyte differentiation were observed via western blotting. (E) Autophagy‐related protein expression was detected by western blotting. (F) Identification of LC3B expression using immunofluorescence; scale bar: 10 μm. *n* = 3 independent experiments. Two‐way ANOVA with the Fisher’s LSD post‐hoc test (B) and student’s *t* test was used for statistical analysis  ^∗^ *p* < 0.05,  ^∗∗^ *p* < 0.01,  ^∗∗∗^ *p* < 0.001 vs. ADSCs.

**Figure:**
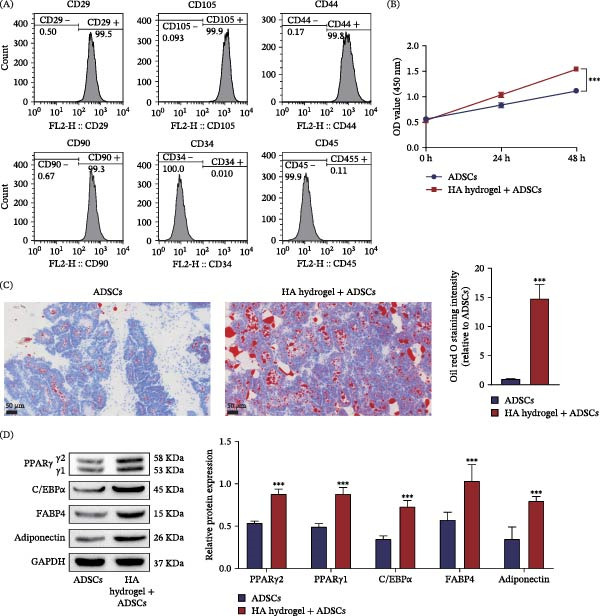

**Figure:**
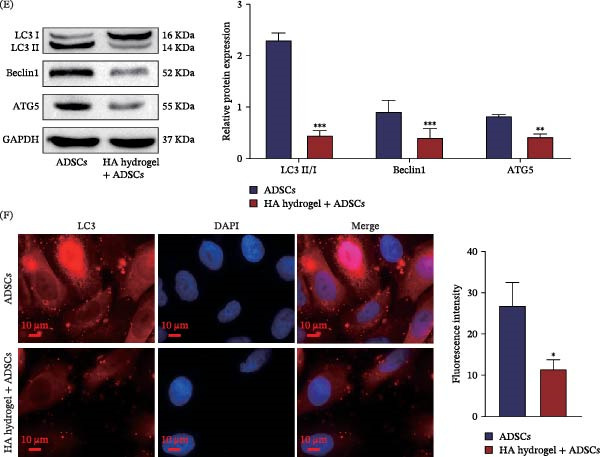

### 3.2. Autophagy Activators Partially Attenuated the Effect of the HA Hydrogel on the Adipogenic Differentiation of ADSCs

**Figure 2:**
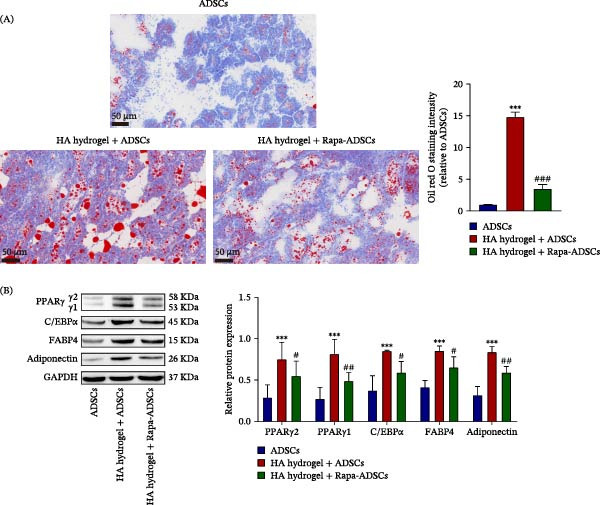
Autophagy activators partially attenuated the effect of the HA hydrogel on the adipogenic differentiation of ADSCs. (A) The capacity of ADSCs to differentiate into adipocytes was identified through Oil Red O staining; scale bar: 50 μm. (B) Western blotting was used to detect the expression of proteins associated with adipocyte differentiation. *n* = 3 independent experiments. One‐way ANOVA with the Fisher’s LSD post‐hoc test was used for statistical analysis.  ^∗∗∗^ *p* < 0.001 vs. ADSCs; ^#^ *p* < 0.05, ^##^ *p* < 0.01 vs. HA hydrogel + ADSCs.

To verify the effect of autophagy on the adipogenic differentiation of ADSCs, 100 nM rapamycin (Rapa), an autophagy activator, was introduced into ADSCs. Oil Red O staining results showed that compared with the HA hydrogel + ADSCs, Rapa markedly reduced the adipogenic differentiation of ADSCs (Figure 2A). Additionally, western blot analysis revealed that the addition of Rapa markedly reduced the levels of PPARγ, C/EBPα, FABP4, and adiponectin (Figure 2B). These findings suggest that activating autophagy can partially reverse the promoting effect of the HA hydrogel on the adipogenic differentiation of ADSCs.

### 3.3. HA Hydrogels Inhibited the Autophagy of ADSCs Through miR‐181a‐5p

The mechanism through which the HA hydrogel affects autophagy in ADSCs was subsequently investigated. An examination of the MiTED database [23] revealed that the expression level of miR‐181a‐5p ranked among the highest 2% in ADSCs (Figure 3A). Additionally, an examination of the EV miRNA database [24] revealed that exosomes derived from hADSCs expressed miR‐181a‐5p. Furthermore, miR‐181a‐5p is associated with autophagy [20]. Therefore, we speculate that the HA hydrogel inhibits the autophagy of ADSCs by influencing miR‐181a‐5p. RT‒qPCR analysis revealed a notable increase in miR‐181a‐5p expression following HA hydrogel and ADSCs coculture compared with that in the ADSCs group (Figure 3B). Moreover, miR‐181a‐5p was suppressed in ADSCs, leading to a notable decrease in miR‐181a‐5p expression in the miR‐181a‐5p inhibitor group (Figure 3C). Next, the levels of proteins related to autophagy were measured. Compared with the HA hydrogel + ADSCs group, the miR‐181a‐5p inhibitor markedly increased the expression of LC3II/I (LC3B), Beclin‐1, and ATG5 (Figure 3D, E). The accumulation of LC3II in the presence of the autophagic flux inhibitor bafilomycin A1 (Baf A1) is considered evidence of autophagic flux [25]. To further detect the changes in LC3II caused by autophagic flux blockade, we treated ADSCs with Baf A1 (200 nM, 2 h). The Western blot results revealed that Baf A1 treatment increased the accumulation of LC3II, indicating impaired autophagic flux. However, HA hydrogel treatment prevented autophagic flux damage in ADSCs (Figure 3F). These findings revealed that the inhibitory effect of the HA hydrogel on the autophagy of ADSCs was realized by enhancing the miR‐181a‐5p expression.

Figure 3HA hydrogels inhibited ADSC autophagy through miR‐181a‐5p. (A) Identification of miRNAs with the highest 2% expression in adipose‐derived stem cells. (B) The expression of miR‐181a‐5p in ADSCs was detected via RT‒qPCR. (C) The transfection efficiency of the miR‐181a‐5p inhibitor in ADSCs was detected via RT‒qPCR. (D) Autophagy‐related proteins LC3II/I, ATG5, and Beclin 1 were detected via western blotting. (E) LC3B expression was detected through immunofluorescence; scale bar: 10 μm. (F) After ADSCs were treated with bafilomycin A1, the level of LC3Ⅱ was detected by western blotting. *n* = 3 independent experiments. Student’s *t* test (B) and one‐way ANOVA with the Fisher’s LSD post‐hoc test was used for statistical analysis.  ^∗^ *p* < 0.05,  ^∗∗∗^ *p* < 0.001 vs. ADSCs or NC inhibitor; ^##^ *p* < 0.01, ^###^ *p* < 0.001 vs. HA hydrogel + ADSCs.

**Figure:**
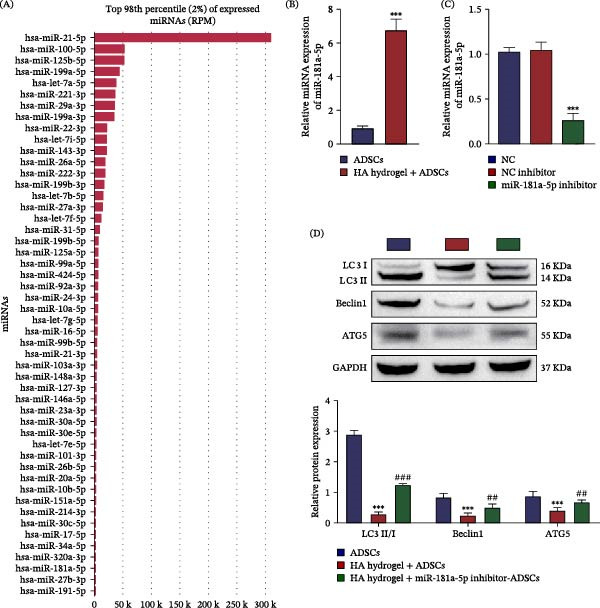

**Figure:**
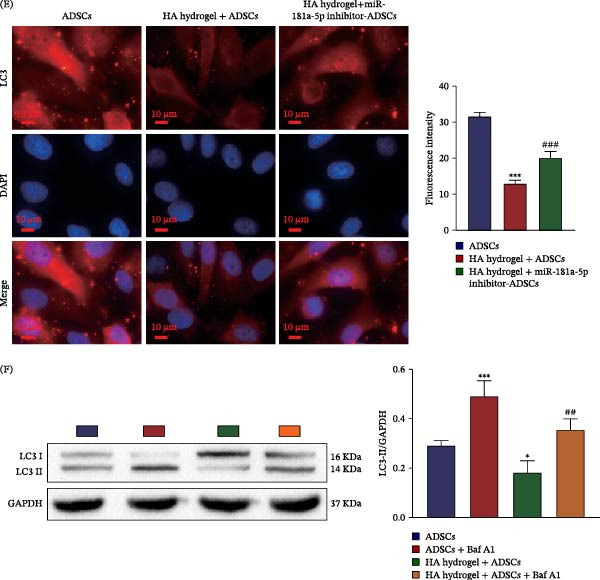

### 3.4. miR‐181a‐5p Targeted the Regulation of ATG5 Expression

**Figure 4:**
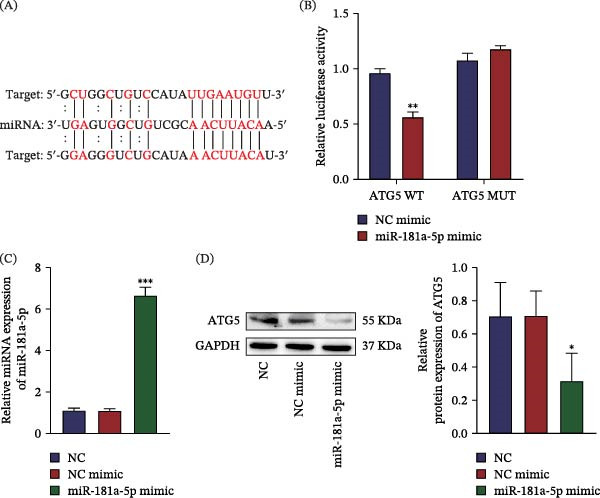
miR‐181a‐5p targets ATG5 expression. (A) The specific binding site for miR‐181a‐5p and ATG5. (B) A dual‐luciferase reporter gene assay was used to verify the binding of miR‐181a‐5p to ATG5. (C) RT‒qPCR analysis of the transfection efficacy of the miR‐181a‐5p mimic in ADSCs. (D) Western blot analysis of ATG5 expression in ADSCs. *n* = 3 independent experiments. Student’s *t* test (B) and one‐way ANOVA with the Fisher’s LSD post‐hoc test was used for statistical analysis.  ^∗∗^ *p* < 0.01,  ^∗∗∗^ *p* < 0.001 vs. NC or NC mimic.

The downstream regulatory mechanism of miR‐181a‐5p was subsequently examined. ATG5 plays a crucial role in controlling autophagy [12], with research indicating that miR‐181a‐5p can regulate the expression of ATG5 [20]. Consequently, through the use of a bioinformatics site (http://starbase.sysu.edu.cn/), we identified the specific binding site of miR‐181a‐5p and found that ATG5 was a downstream target of miR‐181a‐5p (Figure 4A). Additionally, the dual‐luciferase reporter gene assay confirmed that miR‐181a‐5p could target and bind to ATG5 (Figure 4B). Moreover, miR‐181a‐5p was overexpressed in ADSCs, and the expression level of miR‐181a‐5p in the miR‐181a‐5p mimic group was significantly upregulated (Figure 4C). Ultimately, western blot analysis revealed that the overexpression of miR‐181a‐5p significantly inhibited the expression of ATG5 (Figure 4D). These findings suggest that miR‐181a‐5p can target and negatively regulate the expression of ATG5.

### 3.5. Overexpression of ATG5 Attenuated the Effect of the HA Hydrogel on the Adipogenic Differentiation of ADSCs

**Figure 5:**
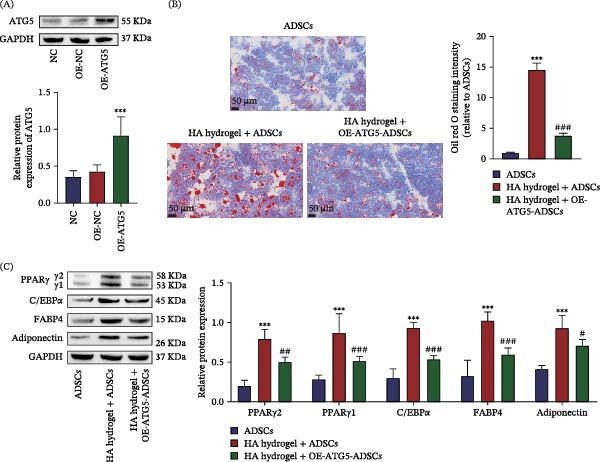
Overexpression of ATG5 attenuated the effect of the HA hydrogel on the adipogenic differentiation of ADSCs. (A) Western blotting was used to assess the effectiveness of OE‐ATG5 transfection in ADSCs. (B) Oil Red O staining was used to evaluate the capacity of ADSCs for adipogenic differentiation; scale bar: 50 μm. (C) Western blotting was used to identify the levels of the adipocyte differentiation‐related proteins PPARγ, C/EBPα, FABP4, and adiponectin. *n* = 3 independent experiments. One‐way ANOVA with the Fisher’s LSD post‐hoc test was used for statistical analysis.  ^∗∗∗^ *p* < 0.001 vs. NC or ADSCs; ^#^ *p* < 0.05, ^###^ *p* < 0.001 vs. HA hydrogel + ADSCs.

To determine the effect of ATG5 on the adipogenic differentiation of ADSCs, we overexpressed ATG5 in ADSCs, and the expression level of ATG5 in the OE‐ATG5 group was significantly increased (Figure 5A). Furthermore, the Oil Red O staining results revealed that, compared with the HA hydrogel + ADSCs group, the ATG5‐overexpressing group showed markedly reduced adipogenic differentiation ability of ADSCs (Figure 5B). Western blot analysis revealed that ATG5 overexpression markedly reduced the levels of PPARγ, C/EBPα, FABP4, and adiponectin compared with those in the HA hydrogel + ADSCs group (Figure 5C). These results indicate that the overexpression of ATG5 can partially reverse the promoting effect of the HA hydrogel on the adipogenic differentiation of ADSCs.

### 3.6. ADSC‐Composite HA Hydrogels Promoted Adipose Tissue Formation In Vivo

**Figure 6:**
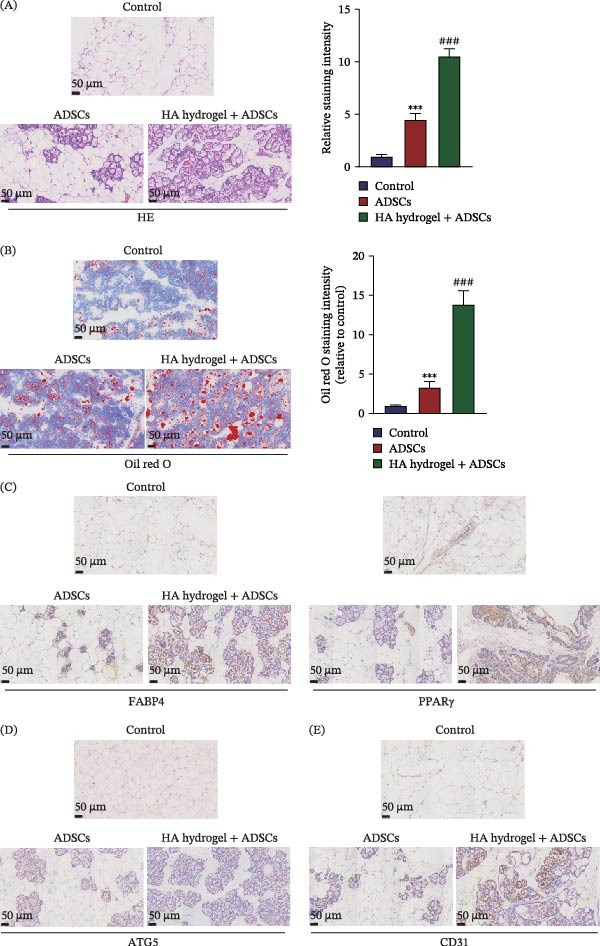
ADSC‐composite HA hydrogel promoted adipose tissue formation in vivo. (A) Morphological changes in adipose tissue from nude mice were observed by HE staining; scale bar: 50 μm. (B) Oil Red O staining was used for adipogenesis detection in nude mice; scale bar: 50 μm. (C) FABP4 and PPARγ expression was measured via immunohistochemical staining; scale bar: 50 μm. (D) Immunohistochemical staining was used to detect the expression of ATG5; scale bar: 50 μm. (E) Immunohistochemical staining was used to detect the expression of CD31; scale bar: 50 μm. *n* = 5 mice per group. One‐way ANOVA with the Fisher’s LSD post‐hoc test was used for statistical analysis.  ^∗∗∗^ *p* < 0.001 vs. Control; ^###^ *p* < 0.001 vs. ADSCs.

ADSCs have low immunogenicity and can reduce rejection reactions in recipient animals [26]. Taking advantage of this property, we injected ADSCs into nude mice. The ability of the HA hydrogel to promote the adipogenic differentiation of ADSCs was subsequently verified in nude mice. HE staining revealed that the HA hydrogel group, which included ADSCs, demonstrated a merging of the adipose tissue with the HA hydrogel, resulting in a structured arrangement (Figure 6A). Compared with the control group, the ADSCs group exhibited notably greater adipogenesis, with the HA hydrogel + ADSCs group showing more pronounced adipogenesis than the ADSCs group did (Figure 6B). Compared with those in the control group, the expression levels of FABP4 and PPARγ in nude mice injected with ADSCs were significantly greater, and the increase in FABP4 and PPARγ expression was more significant after the injection of the HA hydrogel + ADSCs (Figure 6C). In addition, immunohistochemistry was used to detect the expression of the autophagy‐related protein ATG5. The results showed that the injection of ADSCs reduced the expression of ATG5, whereas the injection of the HA hydrogel + ADSCs further reduced the expression of ATG5 (Figure 6D). The CD31 immunohistochemistry (a marker for capillary formation) showed that compared with the control group, there was a small amount of CD31 expression in the ADSCs group, while in the HA hydrogel + ADSCs group, the expression of CD31 was more abundant (Figure 6E), indicating that the HA hydrogel can promote capillary development and enhance the survival of the graft. These results indicate that ADSCs with the HA hydrogel can significantly promote the formation of adipose tissue in vivo.

## 4. Discussion

Research has indicated that adipose tissue engineering technology based on ADSCs, as a means of tissue regeneration and repair reconstruction, is a relatively ideal alternative to traditional transplantation [27–29]. Research has shown that adverse environments, such as ischemia, hypoxia, and local inflammation after transplantation, can aggravate the apoptosis and senescence of ADSCs, weaken their adipogenic differentiation ability, and, to a large extent, limit the positive pro‐regenerative effect of ADSCs during adipose transplantation [30]. Therefore, ADSCs need an appropriate environmental niche to perform their optimal function. The use of scaffold material is one of the three essential core factors of ADSC‐based adipose tissue engineering technology, which can not only promote the ability of seed cells to form new tissues but also provide the necessary microenvironment for seed cell growth [31]. The combination of the HA hydrogel and ADSCs can compensate for the deficiencies of ADSCs. It not only provides a suitable microenvironment for the functional exertion of stem cells but also mediates cell proliferation and maintains cell stemness [32]. Moreover, Lin et al. [33] reported that HA hydrogels are capable of triggering the adipogenic differentiation of adipogenic stromal cells. This research also explored the effects of HA hydrogel on ADSCs and revealed that the combined culture of ADSCs and HA hydrogel significantly promoted the proliferation and adipogenic differentiation ability of ADSCs and promoted the formation of adipose tissue in vivo. This finding is similar to earlier research results, yet the specific regulatory mechanism through which HA hydrogels facilitate the adipogenic differentiation of ADSCs is still not well understood.

Adipose tissue, as an active metabolic organ, plays an important role in maintaining the functional health of mammals. Autophagy is one of the key mechanisms that promotes organ metabolism. The role of autophagy in the adipogenic differentiation of ADSCs has always been controversial. Some studies have shown that autophagy promotes the adipogenic differentiation of ADSCs, whereas others believe that autophagy inhibits the adipogenic differentiation of ADSCs. Morganti et al. [22] revealed that elevated levels of promyelocytic leukemia proteins enhance PKCβ expression, which in turn increases PPARγ by inhibiting autophagy, thereby leading to the adipogenic differentiation of ADSCs. Similarly, Akita et al. [34] revealed that atazanavir can inhibit the adipogenic differentiation of ADSCs by activating autophagy. In contrast, Cruciani et al. [35] revealed that metformin and vitamin D inhibited the adipogenic differentiation of ADSCs through the suppression of autophagy. Therefore, our research also explored the role of autophagy in the adipogenic differentiation of ADSCs. Our data revealed that ADSCs cocultured with HA hydrogel can significantly inhibit the expression of the autophagy‐related proteins LC3Ⅱ/Ⅰ, ATG5, and Beclin‐1, whereas the activation of autophagy inhibited the expression of the adipocyte differentiation‐related proteins PPARγ, C/EBPα, FABP4, and adiponectin and inhibited the adipogenic differentiation ability of ADSCs. The findings of our research are consistent with those of Morganti et al. [22] and Akita et al. [34] but contrary to the findings of Cruciani et al. [35], which are potentially linked to the culture environment, state, and regulatory mode of ADSCs. This study highlights the roles of the HA hydrogel and miR‐181a‐5p in the autophagy and adipogenic differentiation of ADSCs. These cultivation environments and regulatory factors are different from those in previous studies.

Among the factors regulating the adipogenic differentiation and autophagy of ADSCs, many studies have shown that miRNAs not only regulate the occurrence of autophagy but also regulate the adipogenic differentiation process of ADSCs. For example, Wei et al. [36] revealed that increased levels of miR‐20a in ADSCs enhance the healing impact of lupus nephritis in mice by triggering autophagy. Exosomes derived from ADSCs modified with miR‐181‐5p can prevent liver fibrosis by activating autophagy [37]. In addition, Li et al. [38] reported that miR‐30a has the potential to inhibit C8orf4 expression, thereby hindering the adipogenic differentiation of ADSCs. Therefore, our investigation focused on the miRNAs responsible for controlling autophagy and the adipogenic differentiation of ADSCs. An examination of the MiTED database [23] revealed that the expression of miR‐181a‐5p in ADSCs ranked among the highest 2%. Ouyang et al. [19] revealed that miR‐181a‐5p may increase differentiation and fat production in 3T3‐L1 preadipocytes. Consequently, this project focused on miR‐181a‐5p. Our research revealed that the expression of miR‐181a‐5p was increased in ADSCs after coculture with HA hydrogel, and knocking down miR‐181a‐5p promoted the expression of the autophagy‐related proteins LC3Ⅱ/Ⅰ, ATG5, and Beclin‐1. These results suggest that the HA hydrogel can inhibit the autophagy of ADSCs by promoting the expression of miR‐181a‐5p, which may be an important mechanism regulating the adipogenic differentiation of ADSCs.

Additionally, regarding the downstream mechanism through which miR‐181a‐5p regulates autophagy in ADSCs, increasing evidence suggests a link between autophagy in diverse physiological and pathological activities and the regulation of ATG5 [39]. Studies have shown that knocking down FTO reduces the expression of ATG5, thereby inhibiting autophagy and regulating adipogenesis [40]. Crucially, research has indicated that miR‐181a‐5p is capable of suppressing ATG5 expression, thus preventing autophagy triggered by standard HDL [20]. Our research revealed that miR‐181a‐5p has specific binding sites for ATG5, and dual‐luciferase reporter gene assays and Western blot detection confirmed that miR‐181a‐5p can target and negatively regulate the expression of ATG5. The overexpression of ATG5 suppressed the production of PPARγ, C/EBPα, FABP4, and adiponectin while weakening the promoting effect of the HA hydrogel on the adipogenic differentiation of ADSCs. Our data indicate that miR‐181a‐5p inhibits autophagy by suppressing the expression of ATG5, thereby promoting the adipogenic differentiation of ADSCs. Therefore, targeting the miR‐181a‐5p/ATG5 molecular axis can regulate the autophagy and adipogenesis of ADSCs. Furthermore, intracellular signaling pathways are extremely complex. In the future, further exploration of how the miR‐181a‐5p/ATG5 regulatory axis interacts with other signaling pathways to jointly regulate adipogenesis and autophagy is necessary. In addition, the regulatory function of ATG5 in autophagy may be related to mTORC1 and AMPK signaling [41, 42]. These studies have provided us with new research directions regarding the regulation of adipogenesis and autophagy by ATG5.

On the basis of the above analysis, the HA hydrogel shows great potential for promoting the adipogenic differentiation of ADSCs. Studies have shown that delivering miRNA in the HA hydrogel can increase MSC cartilage production and provide an effective minimally invasive treatment method for articular cartilage repair [43]. Furthermore, the HA hydrogel system loaded with miR‐29a exosomes can maintain the activity and half‐life of exosomes, thereby promoting the osteogenic differentiation of BMSCs and accelerating fracture healing [44]. From a clinical perspective, the results of this study provide a theoretical basis for the development of miR‐181a‐5p‐activated hydrogels. HA hydrogel is a safe and widely used biomaterial. Its biocompatibility and structural properties make it promising for inducing the adipogenic differentiation of ADSCs via the delivery of miR‐181a‐5p, providing a new strategy for clinical adipose tissue engineering. In addition, although the application scope of the HA hydrogel is very wide, its long‐term stability in vivo still needs to be evaluated.

In conclusion, our study revealed that the HA hydrogel inhibited the expression of ATG5 by promoting the expression of miR‐181a‐5p, thereby inhibiting autophagy and promoting the adipogenic differentiation of ADSCs. This study confirmed for the first time that the HA hydrogel can regulate the adipogenic differentiation of ADSCs through the miR‐181a‐5p/ATG5 molecular axis. Our study provides new therapeutic strategies and targets for clinical tissue repair and tissue reconstruction. However, this study has certain limitations. First, we did not investigate the role of miR181a‐5p in adipogenesis and autophagy in animals. Additional experiments are still needed in the future to evaluate the effect of miR181a‐5p in vivo. Second, transplanted fat is often weighed in relation to the implanting volume to determine retention/resorption [45]. Based on previous studies [46], we established an animal model. Although the experimental results were in line with our expectations, we still need to evaluate the survival rate/resorption of the grafts, addressing this will be a focus of our future work.

## Author Contributions

Conceptualization: Fengshan Gan and Xian Zhao. Data curation: Fengshan Gan, Fan Zheng, and Qingzhu Zhou. Formal analysis: Fan Zheng, Boyan Liu, and Bin Yang. Funding acquisition: Jia He. Investigation: Fengshan Gan, Lianzhu Ou, and Wenli Huang. Methodology: Fan Zheng and Xinxin Yang. Project administration: Xian Zhao. Resources: Jia He. Software: Zhuo Gong and Yunyu Xiong. Supervision: Jia He. Validation: Fengshan Gan. Visualization: Qingzhu Zhou and Boyan Liu. Writing – Original Draft: Fengshan Gan and Fan Zheng. Writing – Review & Editing: Jia He and Xian Zhao. All the authors contributed substantially to this manuscript.

## Funding

This study was supported by the Yunnan Province Science and Technology Plan Project Kunming Medical University Joint Special Project (202201AY070001‐199), Kunming Spring City Youth Talent Project, and Kunming Health Science and Technology Talent Training Program Medical Science and Technology Discipline Reserve Talents (2022‐SW (Reserve)‐89).

## Disclosure

All the authors have read and approved the final manuscript.

## Ethics Statement

All of the experiments were approved by the Medical Ethics Committee of the First People’s Hospital of Kunming (2025‐032‐01) and performed in accordance with the Declaration of Helsinki, and informed consent was obtained from all participants. All experimental protocols were approved by the Animal Ethics and Welfare Committee of Kunming Medical University (kmmu20230132), and all animal experiments were conducted in accordance with the ARRIVE guidelines.

## Conflicts of Interest

The authors declare no conflicts of interest.

## Data Availability

The data are available from the corresponding author upon reasonable request.
